# Supplementary material for: High Piezoelectric Output Voltage from Blue Fluorescent N,N-Dimethyl-4-nitroaniline Nano Crystals in Poly-L-Lactic Acid Electrospun Fibers
Source: Materials (Basel). 2022 Nov 10;15(22):7958. doi: 10.3390/ma15227958 (PMC9698555; doi:10.3390/ma15227958)
Supplement: Supplementary file 1 [file materials-15-07958-s001.zip › materials-1958370-supplementary.pdf]

High Piezoelectric Output Voltage from Blue Fluorescent *N,N*-dimethyl-4-nitroaniline Nano Crystals in poly-l-lactic Acid Electrospun Fibers

Rosa M. F. Baptista, Bruna Silva, João Oliveira, Vahideh B. Isfahani, Bernardo Almeida, Mário R. Pereira, Nuno Cerca, Cidália Castro, Pedro V. Rodrigues, Ana Machado, Michael Belsley and Etelvina de Matos Gomes

Suplemmentary Information

SI 1.1. *X-ray Diffraction*

The crystallinity and crystallographic orientation of *N,N*-dimethyl-4-nitroaniline (NNDM4NA) was studied by X-ray diffraction. The diffraction pattern using θ–2θ scans was recorded between 5º and 40º on a Philips PW-1710 X-Ray diffractometer with Cu-K$_\alpha$ radiation of wavelength 1.5406 $\mathring{A}$. The lattice planes parallel to the substrate surface were determined from the reciprocal lattice vector of the modulus $(2/\lambda)\sin\theta$, with λ the radiation wavelength and θ the Bragg angle. The X-ray diffraction pattern shows that NNDM4NA is crystallized inside the poly L-latic acid (PLLA) [1] polymer matrix with random orientation.

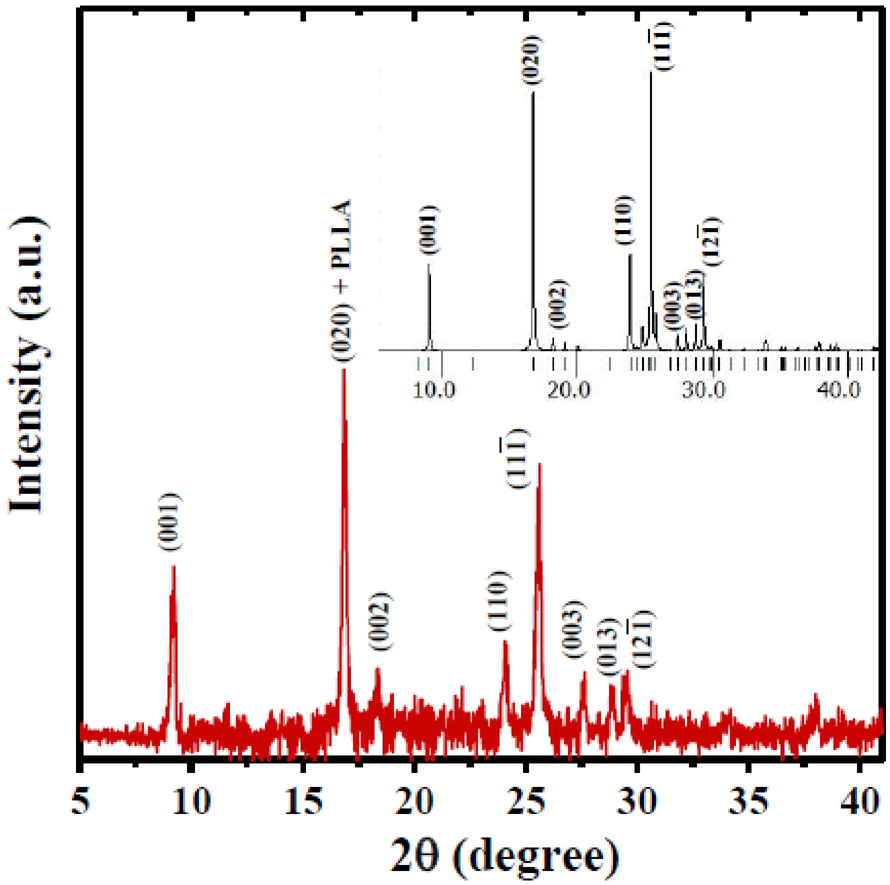

Figure S1. The measured X-ray pattern of *N,N*-dimethyl-4-nitroaniline (NNDM4NA) embedded into polymer fibers, the inset shows the calculated X-ray pattern of N,N-dimethyl-4-nitroaniline (NNDM4NA) using the published crystallographic information (cif file).

By using the Debye–Scherrer equation [2], the crystallite size of NNDM4NA is given by [3]

$$t = \frac{K\lambda}{\beta_{hkl} \cos\theta_{hkl}} \quad (1)$$

In this equation, the parameter K is a shape factor which we take to be 0.90, while λ is the X-ray wavelength. The full-width half-maximum of a reflection (hkl) is β_{hkl}. This width was estimated using LIPRAS Line Profile Analysis Software [3] to fit an asymmetric Pseudo Voight Profile to and peaks. Both fits lead to an average crystallite size of 38 nm. The relevant fit parameters are listed in Table S1, with the corresponding fits shown in Figure S2 a) and b). (020) (11 1)

Table S1. Crystallite size and fit parameter values for (020) and (11 1) Bragg reflections.

	$2\theta_{hkl}$	β_{hkl}	t
(020)	16.871º	0.212º	39.5 nm
(11 1)	25.583º	0.229º	37.2 nm

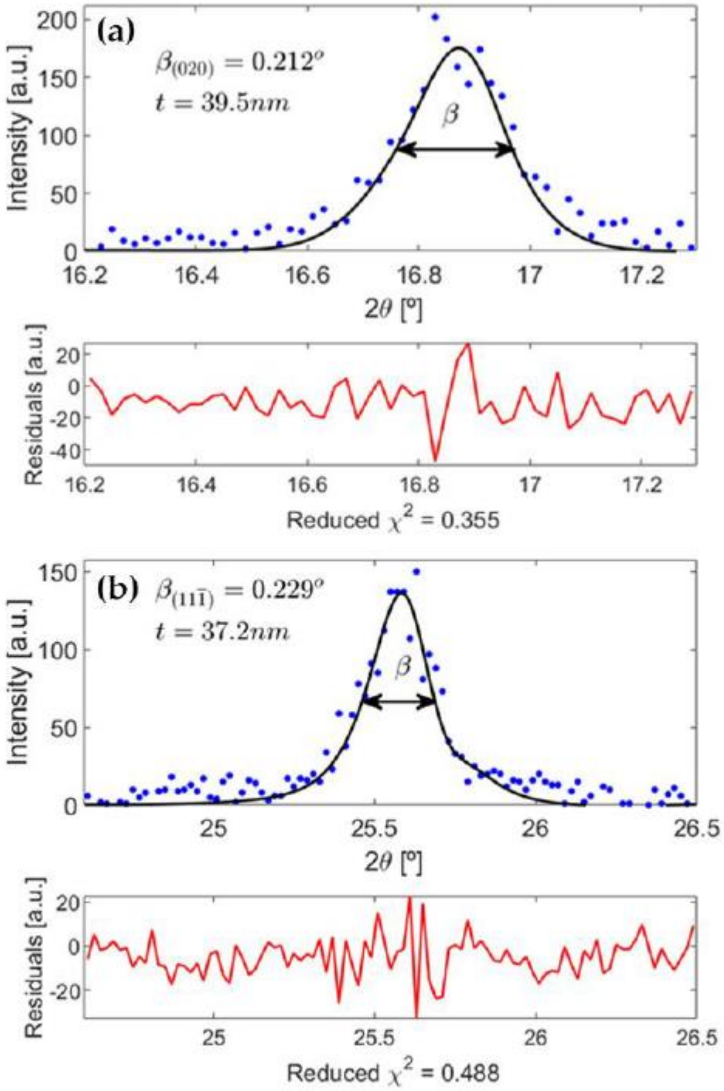

Figure S2. Asymmetric Pseudo-Voigt fits of (020) and (11 1) Bragg reflections for NNDM4NA. The width of the reflections at half the maximum β and the average size of the calculated nanocrystals t is indicated.

SI 1.2. *Confocal Laser Scanning Microscopy*

The autofluorescence of the fibers was observed with an Olympus™ FluoView FV1000 (Olympus, Tokyo, Japan) confocal scanning laser microscope, using a 40x objective, with several emission/detection settings: (i) excitation wavelength 405 nm, detection filters BA 430–470; BA 505-
3
540; BA 480-495; (ii) excitation wavelength 458 nm, detection filters BA 505-605; BA 575-620; BA 535-565 or (iii) excitation wavelength 559 nm, detection filters BA 575-675; BA 655-755. Images were acquired with 800 x 800-pixel resolution. A 1 cm_2 fiber mat with 600 µm thickness was observed on a glass slide. A scan of the sample was performed at room temperature.

SI 1.3. *Fluorescence lifetime decay*

The fluorescence lifetime was measured using a single-photon counting system equipped with a NanoLed® as an excitation light source (Horiba, Jovin-Yvon, IBH Ltd.) with peak emission at 370 nm

and an impulse repetition rate of 1 MHz. The excitation wavelength was selected using a monochromator, and the fluorescence emission was selected using filters and detected by a photomultiplier (Hamamatsu R2949). The counting acquisition was made using a Becker&Hickl SPC-150 TCSPC board, and data analysis was performed using PicoQuant FluoFit software. A multi-exponential model was used to estimate the fluorescence lifetimes,

$$I(t) = \sum_i^n \alpha_i exp\left(-\frac{t}{\tau_i}\right) \quad (2)$$

where $I(t)$ is the intensity as a function of time, α_i are the amplitudes and τ_i are the decay lifetimes (ns). The goodness of fit was assessed in terms $\chi 2$ residuals and autocorrelation function analysis. Each fluorescence fraction, f_i, was determined using the equation,

$$f_i = \frac{\alpha_i \tau_i}{\sum_j^n \alpha_i \tau_i} \quad (3)$$

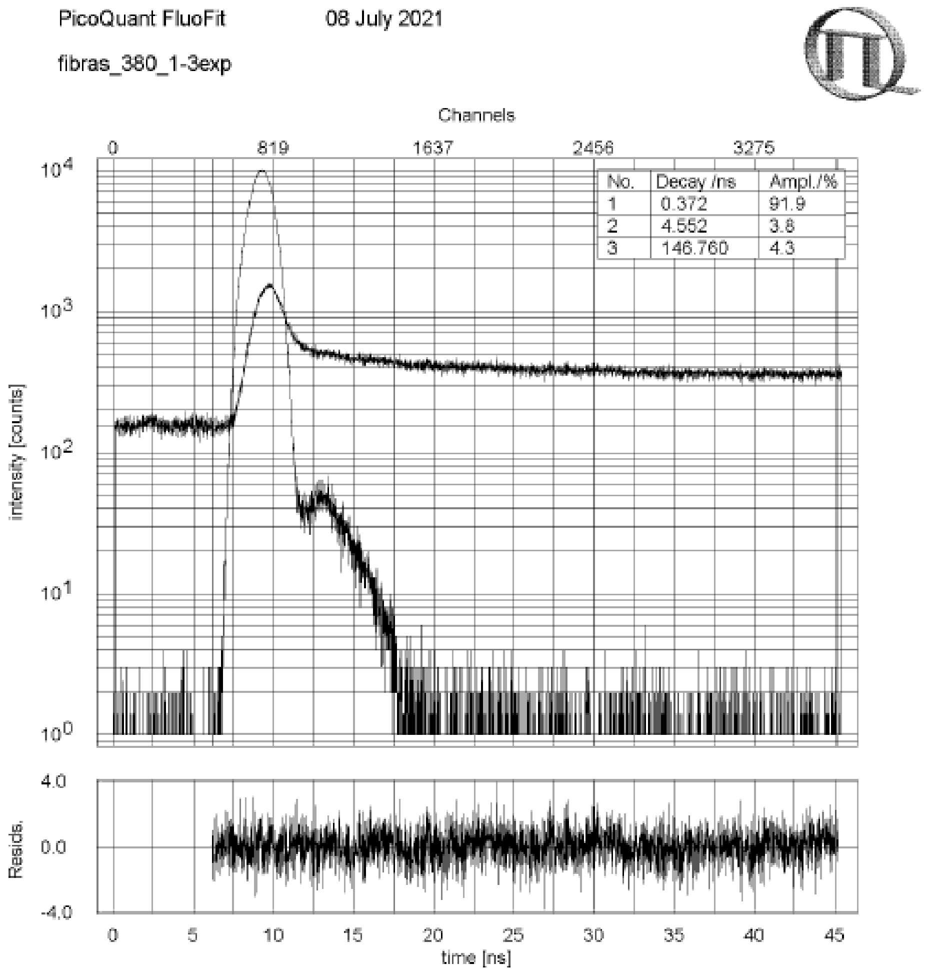

Fitting function: 3 - exponential, reconvolution

No.	Decay / ns	Error Decay	Amplitude	rel. Amplitude
1	0.372	0.013	5528.324	91.9 %
2	4.552	0.277	229.924	3.8 %
3	146.757	11.805	255.705	4.3 %
Av.	135.688			

X²(reduced): 1.018
Background Sample: 152.79
IRF: 1.00
IRF Shift / channel : -9.26

Figure S3. Fluoresce lifetime decay measured using a single photon counting system equipped with a NanoLed® as excitation light source (Horiba, Jovin-Yvon, IBH Ltd.) with peak emission at 370 nm and an impulse repetition rate of 1 MHz. 4

References

1. Wasanasuk, K.; Tashiro, K.; Hanesaka, M.; Ohhara, T.; Kurihara, K.; Kuroki, R.; Tamada, T.; Ozeki, T.; Kanamoto, T. Crystal Structure Analysis of Poly(l-lactic Acid) α Form On the basis of the 2-Dimensional Wide-Angle Synchrotron X-ray and Neutron Diffraction Measurements. *Macromolecules* **2011**, 44, 6441-6452.
2. Hammond, C. *The Basics of Crystallography and Diffraction*, 3rd ed.; International Union of Crystallography Texts on Crystallography: Chester, UK, 2015.
3. Esteves, G.; Ramos, K.; Fancher, C.M.; Jones, J.L. *LIPRAS: Line-Profile Analysis Software*; MathWorks: Natick,MA, USA, 2017.
